# Supplementary material for: Rural-urban disparities in nutritional status among ever-married women in Bangladesh: A Blinder-Oaxaca decomposition approach
Source: PLoS One. 2023 Dec 14;18(12):e0289880. doi: 10.1371/journal.pone.0289880 (PMC10721169; doi:10.1371/journal.pone.0289880)
Supplement: Attachment — Submitted filename: Author Response (2).docx [file pone.0289880.s001.docx]

**Additional Editor Comments:**
Your manuscript writing quality are not well standard. You need to improve the quality of your paper. Also need to proof read your paper by a English native person.

Author Response: Thank you for your suggestions. We have revised our English language with the help of an expert member.

Dear Editor,

On behalf of all the authors, I wish to convey our gratitude to you for the critical and constructive review that has led to the improvement of our manuscript entitled ***“Health policy improvement via inequality assessment in the nutritional status of ever-married women in Bangladesh: A decomposition analysis.”*** We would like to thank the editor and the reviewers for their useful feedback. We have revised the manuscript based on the comments raised by the reviewer. We believe the manuscript has improved according to reviewer comments and its English level substantively and will be published in your reputable Journal, ***“PLOS ONE.”*** All the changes are highlighted in the yellow color in the revised manuscript.

Thanks and Best regards

Iqramul Haq

Corresponding author

**##** **The following is a point-by-point response to the reviewer(s) comments:**

**Reviewer #1:**

*1. Define explanatory variable in method section and explain how you collect them.*

**Response: We make necessary changes in section 2.4 in the revised manuscript.**

2. How you calculate economic variable?

**Response: We added about economic variable details in section 2.4 as: The authority of Bangladesh Demographic and Health Survey provides a wealth index indicator that is a composite measure of a household's cumulative living standard and is calculated using information about a household's ownership of selected assets: televisions and bicycles; materials used for housing construction; and types of water access and sanitation facilities. For detailed information on how the wealth score is calculated, see the survey report from “https://dhsprogram.com/publications/publication-FR344-DHS-Final-Reports.cfm”.**

*3. You define malnutrition as BMI<18.5 vs BMI>18.5, is your it includes overweight and obesity too (which is malnutrition themselves), please clarify your outcome is malnutrition or just include underweight.*

**Response: This is an unintentional mistake. We mainly focus on under nutrition part and coded as 1 in dependent variable.**

*4. It is not necessary to bring all the table’s contents in the text, just mention main results in table 1 and 2.*

**Response: We make necessary changes in explanation of Table 1 and 2 in the revised manuscript.**

*5. In table 5 please specify which determinants are significant.*

**Response: We added significant sign in Table 5 in the revised manuscript.**

**Reviewer #2:**

*1. The manuscript has explored an interesting topic of malnutrition which is highly prevalent in Bangladesh. The introduction seems to be missing points on why such analysis or geographic inequality is important in the context of Bangladesh? Please don’t provide description of the analysis in the introduction. It needs a little more context and motivation for performing the analysis.*

**Response: We make necessary changes in introduction section in revised manuscript.**

*2. The study is only looking at the undernutrition among women (BMI<18.0). Author need to focus on that. After reading the introduction it feels like author will be exploring both underweight and overweight among women which is not the case.*

**Response:** **This is an unintentional mistake. We mainly focus on under nutrition part and coded as 1 in dependent variable.**

*3. We can see a high prevalence of undernutrition the rural areas, more focus should be on that rather than providing generic statements in the introduction.*

**Response: We made necessary changes as per reviewer suggestions in introduction section in the revised manuscript.**

*4. I will request author to avoid sentences like this is only study that provide these estimates. Rather than emphasize on why such study needed and contribute to literature or policy level decisions.*

**Response: We have added in our revised manuscript.**

***Tables and graphs***

*5. I am not able to understand the need of figure 2. If you can provide this figure separately for rural and urban areas, it will make more sense.*

**Response: We added urban and rural concentration index as tabulation formation.**

*6. I will request to provide either confidence interval or p-values in the table 5. Author also needs to provide percentages. What percentage were explained by endowments in the table itself.*

**Response: We added significant sign in Table 5 in the revised manuscript. We also added the endowments percentage in Table 4 explanation**

***Discussion and Conclusion***

*7. Discussion needs to strengthen. For example, why mass media is highly associated with underweight. Does the wealth quintile is also associated with multimedia access. Explanation and discussion of the results section is required. Repeating the same that it is one of only study and blinder oxaca decomposition in introduction, discussion and conclusion is not needed. Instead, a more clear explanation of the results and providing few implication of the finding will be very helpful for the readers. Discussion section seems incomplete, it should be rewritten with focus on the findings of the manuscripts.*

**Response: We have revised our updated manuscript.**

##I hope our efforts satisfy the requirements of the journal this time. I will be looking forward to your positive response. Thanks, and regards.
