# Supplementary material for: Rural-urban disparities in nutritional status among ever-married women in Bangladesh: A Blinder-Oaxaca decomposition approach
Source: PLoS One. 2023 Dec 14;18(12):e0289880. doi: 10.1371/journal.pone.0289880 (PMC10721169; doi:10.1371/journal.pone.0289880)
Supplement: Attachment — Submitted filename: comment to authors.docx [file pone.0289880.s002.docx]

Title:

The big problem in policy development of developing countries is since imported or copied from others developed countries. Hence, I as a professional appreciated such root-based data analysis and explaining of the ground level problems. Its good title

Abstract:

Generally it was good if the authors used three or more years survey data. The two years data is some what not explanatory for policy change

On line three of the abstract and in some parts of the main text the authors used malnutrition and undernutrition interchangeably. However, the two health conditions are completely different. Since the title indicated as nutritional status, better to use it instead.

Back ground

Good flow of idea, but close it with your general objective

**Methods and materials**

Univariate, bivariate, and multivariate analysis

What analysis methods you did for univariate? Hope it’s good if omitted

Plus, the authors used “multivariate” analysis which is not inline with their analysis method. In statistics multivariate and multivariable are different. Please read more and replace the “multivariate” by “multivariable”.

Software

What was the advantage of using multiple softwares?, but STATA can perform all the tasks you mentioned. Or one of the can do it. Reduce the junk use of softwares

Results

The logical flow of ideas is that Table 1 information should come first of Figure 1 information

On contribution of each covariates in urban-rural gap, the authors included non-married(see table 5) women in the analysis. But the study populations are ever married. So how could you include?

Decision: since the study will have imputes on the policy development, I recommend to be published
