# Supplementary material for: Rural-urban disparities in nutritional status among ever-married women in Bangladesh: A Blinder-Oaxaca decomposition approach
Source: PLoS One. 2023 Dec 14;18(12):e0289880. doi: 10.1371/journal.pone.0289880 (PMC10721169; doi:10.1371/journal.pone.0289880)
Supplement: Attachment — Submitted filename: Review_Report.docx [file pone.0289880.s003.docx]

General Comment

Thank you for allowing me to review the manuscript. After reviewing the manuscript, found many redundancy. I would suggest the authors carefully go through these and address these. I hope after addressing both the general and specific comments, the quality of the manuscript will be improved. Review without line numbers is tough and boring.

Specific comments

Below are the specific comments—

1. Title: Health Policy Improvement via inequality assessment in the nutritional status.. It seemed to me that due to the inequality assessment, Health Policy has improved. Health policy improvement can be the recommendation from the inequality assessment but should not be in the title. The article title should be based on the study objective. I would suggest the authors critically think about the issue and revise the title based on the study objective.
2. A paper titled “Socioeconomic Inequalities in Women’s Undernutrition: Evidence from Nationally Representative Cross-Sectional Bangladesh Demographic and Health Survey 2017–2018” was published in IJERPH with similar findings. <https://www.mdpi.com/1660-4601/19/8/4698> The authors should mention what will be the added values of this current research. Otherwise, it will be duplicated.
3. In the abstract, “The multivariate analysis on the determinants of undernutrition among ever-married women regarding residence discovered a significant impact….”. Can we say impact in a cross-sectional study? Authors are advised to use an appropriate term.
4. In the background, “Various studies show that the majority of women die from malnutrition….” The authors should cite references here and also need to mention the actual estimates instead of using “Majority”.
5. In Bangladesh, policymakers tried to recover the malnourishment among reproductive aged women [10]…. This sentence does not fit here. You are stating estimates here. Just provide the estimates only. Also mentioned the “Recent studies based on Bangladesh Demographic and Health Survey reported that 12 percent..” It is similar to your study findings. Better to mention the knowledge gaps in this study and your study addressed these knowledge gaps.
6. “According to 2021 global hunger index information, Bangladesh ranks 76th out of 116 countries in the Global Hunger Index”.. Before this sentence, the authors added two sentences which doesn’t match the flow of the writing.
7. In Statistical methods “various independent variables, which were selected by studying previous literature…………..”. Redundancy, previously mentioned. Please avoid redundancy.
8. In the multivariable setup, the effect of independent variables …. Can we say effect? It’s a cross-sectional study.
9. “The odds ratio with a 95% confidence interval was usually used to explain the impact of predictor variables………..” again the term impact, the effect should not be used in a cross-sectional study.
10. This study also measures the socio-economic inequality, and a popular index measure
    named "Concentration index" are used ……………..please check the grammar.
11. This study evaluates the gap due to the i. Difference in the characteristics (E) ii. Difference in the effect of the coefficient (C), and iii. Interaction (CE)……….redundancy. Already mentioned in the method section.
12. Discussion: The results of this study, based on data from a nationally representative cross-sectional survey, show that there are socioeconomic inequalities in the prevalence of malnutrition…. Instead of malnutrition the authors should use undernutrition in all the places. Since malnutrition typically means both under (undernutrition) and over (overweight or obesity) nutrition.
13. Discussion: A double burden was created by the continued high prevalence of underweight status in rural areas… why the author is discussing the double burden? Since this paper is based on undernutrition, the author should discuss only the undernutrition issue.
14. As physical changes gradually increase with age, and due to rapid physical growth, physiological activities increase and require more energy to meet the increasing demands…. Please cite the paper for this statement.
15. (i.e., listened to the radio, watched television, and read the newspaper at least once a week)…. Redundancy. already mentioned in the method section
16. Regular TV viewing has also been linked to an increased risk of obesity and being overweight. Bangladeshi women's weight increases because they are less physically active and spend more time sitting down……………….again obesity issue. It is not this study issue. Please drop Regular TV………to mental health [37, 38].
17. due to the cross-sectional nature of the study, it was not possible to determine the causal-effect relationship.. But in many places, the authors mentioned effects even also impact which is not right for a cross-sectional study. Please carefully mention the term.. simply mention “association”.
18. Conclusion: no need to mention the dataset name and year again previously mentioned many times.
