# Supplementary material for: Rural-urban disparities in nutritional status among ever-married women in Bangladesh: A Blinder-Oaxaca decomposition approach
Source: PLoS One. 2023 Dec 14;18(12):e0289880. doi: 10.1371/journal.pone.0289880 (PMC10721169; doi:10.1371/journal.pone.0289880)
Supplement: Attachment — Submitted filename: Response (8).docx [file pone.0289880.s004.docx]

Dear Editor,

On behalf of all the authors, I wish to convey our gratitude to you for the critical and constructive review that has led to the improvement of our manuscript entitled ***“*Rural-urban disparities in nutritional status among ever-married women in Bangladesh: A Blinder-Oaxaca decomposition approach.”** We have revised the manuscript based on the comments raised by the reviewers. We believe the manuscript has improved according to reviewers comments and its English level substantively and will be published in your reputable Journal, ***“PLOS ONE.”*** All modifications have been marked and highlighted using the track changes feature in the revised manuscript. I hope our efforts satisfy the requirements of the journal this time. I will be looking forward to your positive response. Thanks, and regards.

On the behalf of authors

Iqramul Haq

Corresponding author

**##** **The following is a point-by-point response to comments:**

General Comment

Thank you for allowing me to review the manuscript. After reviewing the manuscript, found many redundancy. I would suggest the authors carefully go through these and address these. I hope after addressing both the general and specific comments, the quality of the manuscript will be improved. Review without line numbers is tough and boring.

**Response: We appreciate your valuable suggestions, and we have incorporated your comments into the revised version of the manuscript.**

Specific comments

Below are the specific comments—

1. Title: Health Policy Improvement via inequality assessment in the nutritional status. It seemed to me that due to the inequality assessment, Health Policy has improved. Health policy improvement can be the recommendation from the inequality assessment but should not be in the title. The article title should be based on the study objective. I would suggest the authors critically think about the issue and revise the title based on the study objective.

**Response: Thank you for your suggestion and feedback regarding the article title. We appreciate your insight on aligning the title with the study objective. After careful consideration, we have revised the title based on your recommendation.**

**Revised Title: "Rural-Urban Disparities in Nutritional Status Among Ever-Married Women in Bangladesh: A Blinder-Oaxaca Decomposition Analysis"**

**We believe that this revised title accurately reflects the study objective of examining rural-urban disparities in nutritional status among ever-married women in Bangladesh and the methodology used in the study. Thank you for your valuable input, and we have incorporated your feedback to enhance the clarity and focus of the title.**

2. A paper titled “Socioeconomic Inequalities in Women’s Undernutrition: Evidence from Nationally Representative Cross-Sectional Bangladesh Demographic and Health Survey 2017–2018” was published in IJERPH with similar findings. https://www.mdpi.com/1660-4601/19/8/4698 The authors should mention what will be the added values of this current research. Otherwise, it will be duplicated.

**Response: Thank you for your valuable feedback on our article. We appreciate your concern about potential duplication with a previously published paper titled "Socioeconomic Inequalities in Women's Undernutrition: Evidence from Nationally Representative Cross-Sectional Bangladesh Demographic and Health Survey 2017-2018" in the International Journal of Environmental Research and Public Health (IJERPH).**

**While the paper you mentioned does share a similar focus on socioeconomic inequalities in women's undernutrition in Bangladesh, our research offers several distinct contributions and advances the existing literature in several important ways. We would like to highlight the added value of our current study, which sets it apart from the previously published work:**

**Focus on Determinants: Unlike the previous study, our research explicitly investigates the determinants of women's undernutrition in the context of urban-rural disparities.**

**Socioeconomic Inequality Analysis: Our study incorporates a comprehensive analysis of socioeconomic inequalities, specifically examining the disparities in undernutrition between different socioeconomic groups within both urban and rural areas, while the previous study you mentioned did not specifically address this aspect. By utilizing established measures of inequality, such as concentration index, we shed light on the magnitude and nature of these disparities.**

**Blinder-Oaxaca Decomposition: To better comprehend the factors driving the observed urban-rural disparities in women's undernutrition, our research employs the Blinder-Oaxaca decomposition technique. This method allows us to disentangle the effects of various factors contributing to the disparities, thereby providing a nuanced understanding of the underlying drivers. Most importantly the previous study you mentioned did not specifically address this aspect.**

**By emphasizing these distinctive aspects, our study significantly extends the existing knowledge on socioeconomic inequalities in women's undernutrition in Bangladesh. We believe that our findings have important implications for policy and intervention strategies aimed at addressing this critical issue.**

**We appreciate your suggestion to explicitly mention the added values of our research in the article. We will revise the manuscript accordingly to ensure that readers are aware of the unique contributions our study brings to the field. Once again, we thank you for your insightful feedback and for helping us enhance the quality and originality of our work.**

3. In the abstract, “The multivariate analysis on the determinants of undernutrition among ever-married women regarding residence discovered a significant impact….”. Can we say impact in a cross-sectional study? Authors are advised to use an appropriate term.

**Response: Considering the inherent limitations of a cross-sectional study design, we acknowledge that causal relationships cannot be established, and therefore, the term "impact" may be misleading. We appreciate your guidance in choosing a more suitable term that aligns with the nature of our study. In light of this, we propose revising the sentence in the abstract as follows:**

**" The multivariable analysis investigating the determinants of undernutrition status among ever-married women, with a focus on residence revealed significant associations with respondent age, education, marital status, mass media access, wealth status, and division."**

4. In the background, “Various studies show that the majority of women die from malnutrition….” The authors should cite references here and also need to mention the actual estimates instead of using “Majority”.

**Response: We have revised this section in the introduction of our manuscript, and it now includes a new citation to provide additional support for the stated information.**

5. In Bangladesh, policymakers tried to recover the malnourishment among reproductive aged women [10]…. This sentence does not fit here. You are stating estimates here. Just provide the estimates only. Also mentioned the “Recent studies based on Bangladesh Demographic and Health Survey reported that 12 percent..” It is similar to your study findings. Better to mention the knowledge gaps in this study and your study addressed these knowledge gaps.

**Response: We have revised this section in the introduction of our manuscript.**

6. “According to 2021 global hunger index information, Bangladesh ranks 76th out of 116 countries in the Global Hunger Index”.. Before this sentence, the authors added two sentences which doesn’t match the flow of the writing.

**Response: We have revised this section in the introduction of our manuscript.**

7. In Statistical methods “various independent variables, which were selected by studying previous literature…………..”. Redundancy, previously mentioned. Please avoid redundancy.

Response: **Response: We have revised this section in the methodology of our manuscript.**

8. In the multivariable setup, the effect of independent variables …. Can we say effect? It’s a cross-sectional study.

**Response: Thank you for your feedback on our article. We appreciate your concern regarding the use of the term "effect" in the context of a cross-sectional study. We understand that cross-sectional studies cannot establish causal relationships and that the term "effect" may be misleading. To address this concern, we will revise the sentence as follows:**

**"In the multivariable setup, the association between independent variables and the nutritional status among ever-married women can be examined using binary logistic regression."**

9. “The odds ratio with a 95% confidence interval was usually used to explain the impact of predictor variables………..” again the term impact, the effect should not be used in a cross-sectional study.

**Response: Thank you for your feedback on our article. We appreciate your concern regarding the use of the term "impact" in the context of a cross-sectional study. We understand that cross-sectional studies cannot establish causal relationships and that the term "impact" may be misleading. To address this concern, we will revise the sentence as follows:**

**"The odds ratio with a 95% confidence interval was usually used to examine the association of predictor variables with the nutritional status."**

10. This study also measures the socio-economic inequality, and a popular index measure

named "Concentration index" are used ……………..please check the grammar.

**Response: We have revised this part in the section 2.5.2 of our revised manuscript.**

11. This study evaluates the gap due to the i. Difference in the characteristics (E) ii. Difference in the effect of the coefficient (C), and iii. Interaction (CE)……….redundancy. Already mentioned in the method section.

**Response: We apologize for any confusion caused by the repetition of this information. Our intention was to provide a simple overview of the Blinder-Oaxaca decomposition in the introduction to give readers an initial understanding of the method. However, a more detailed explanation is provided in the methodology section.**

12. Discussion: The results of this study, based on data from a nationally representative cross-sectional survey, show that there are socioeconomic inequalities in the prevalence of malnutrition…. Instead of malnutrition the authors should use undernutrition in all the places. Since malnutrition typically means both under (undernutrition) and over (overweight or obesity) nutrition.

**Response: We have revised this part in the discussion of our revised manuscript.**

13. Discussion: A double burden was created by the continued high prevalence of underweight status in rural areas… why the author is discussing the double burden? Since this paper is based on undernutrition, the author should discuss only the undernutrition issue.

**Response: We have removed this part from the discussion of our revised manuscript.**

14. As physical changes gradually increase with age, and due to rapid physical growth, physiological activities increase and require more energy to meet the increasing demands…. Please cite the paper for this statement.

**Response: We have removed this part from the discussion of our revised manuscript because of inconsistency.**

15. (i.e., listened to the radio, watched television, and read the newspaper at least once a week)…. Redundancy. already mentioned in the method section

**Response: We have revised this part in the discussion of our revised manuscript.**

16. Regular TV viewing has also been linked to an increased risk of obesity and being overweight. Bangladeshi women's weight increases because they are less physically active and spend more time sitting down………………. again, obesity issue. It is not this study issue. Please drop Regular TV………to mental health [37, 38].

**Response: We have removed this part from the discussion of our revised manuscript because of inconsistency.**

17. due to the cross-sectional nature of the study, it was not possible to determine the causal-effect relationship. But in many places, the authors mentioned effects even also impact which is not right for a cross-sectional study. Please carefully mention the term. simply mention “association”.

**Response: We have revised this part in the discussion of our revised manuscript.**

18. Conclusion: no need to mention the dataset name and year again previously mentioned many times.

**Response: We have revised this part in the discussion of our revised manuscript.**
